# Calculation of Aortic VAlve and LVOT Areas by a Modified Continuity Equation Using Different Echocardiography Methods: The CAVALIER Study

**DOI:** 10.3390/diagnostics12071656

**Published:** 2022-07-07

**Authors:** Tobias Friedrich Ruf, Béatrice Elise Cannard, Ruth H. Strasser, Axel Linke, Krunoslav M. Sveric

**Affiliations:** 1 Center for Cardiology, Cardiology I, University Medical Center Mainz, 55131 Mainz, Germany; 2 Department of Anesthesiology, University Hospital Dresden, Technische Universität Dresden, 01069 Dresden, Germany; becannard@gmx.de; 3 Department of Medicine and Cardiology, Heart Centre Dresden, University Hospital Dresden, Technische Universität Dresden, 01069 Dresden, Germany; ruth.strasser@mailbox.tu-dresden.de (R.H.S.); axel.linke@herzzentrum-dresden.com (A.L.); krunoslav.sveric@mailbox.tu-dresden.de (K.M.S.)

**Keywords:** 3D echocardiography, LVOT, aortic valve area, continuity equation

## Abstract

Background: The area of the left ventricular outflow tract (A_LVOT_) represents a major component of the continuity equation (CE), which is, i.a., crucial to calculate the aortic valve (AV) area (A_AV_). The A_LVOT_ is typically calculated using 2D echo assessments as the measured anterior–posterior (a/p) extension, assuming a round LVOT base. Anatomically, however, usually an elliptical shape of the LVOT base is present, with the long diameter extending from the medial–lateral axis (m/l), which is not recognized by two-dimensional (2D) echocardiography. Objective: We aimed to compare standard and three-dimensional (3D)-echocardiography-derived A_LVOT_ calculation and its use in a standard CE (CE_std_) and a modified CE (CE_mod_) to calculate the A_AV_ vs. computed tomography (CT) multi-planar reconstruction (MPR) measurements of the anatomical A_LVOT,_ and A_AV_, respectively. Methods: Patients were selected if 3D transthoracic echocardiography (TTE), 3D transesophageal echocardiography (TEE), and cardiac CT were all performed, and imaging quality was adequate. The A_LVOT_ was assessed using 2D calculation, (a/p only), 3D-volume MPR, and 3D-biplane calculation (a/p and m/l). A_AV_ was measured using both CE_std_ and CE_mod_, and 3D-volume MPR. Data were compared to corresponding CT analyses. Results: From 2017 to 2018, 107 consecutive patients with complete and adequate imaging data were included. The calculated A_LVOT_ was smaller when assessed by 2D- compared to both 3D-volume MPR and 3D-biplane calculation. Calculated A_AV_ was correspondingly smaller in CE_std_ compared to CE_mod_ or 3D-volume MPR. The A_LVOT_ and A_AV_, using data from 3D echocardiography, highly correlated and were congruent with corresponding measurements in CT. Conclusion: Due to the elliptic shape of the LVOT, use of measurements and calculations based on 2D echocardiography systematically underestimates the A_LVOT_ and dependent areas, such as the A_AV_. Anatomically correct assessment can be achieved using 3D echocardiography and adapted calculations, such as CE_mod_.

## 1. Introduction

Echocardiographic assessment of the left ventricular outflow tract (LVOT) area (A_LVOT_) using transthoracic (TTE) or transesophageal echocardiography (TEE) is a key component for the calculation of various hemodynamic states, such as stroke volume [1], cardiac output and index [2,3,4], and—most crucially—the calculation of the aortic valve (AV) area (A_AV_) [5,6]. The underlying principle is the theorem of a steady flow system in communicating conduits. Here, the laminar flow through the conduit is equal to the product of the mean velocity times the cross-sectional area of the conduit, with $\mathrm{SV}\;={\;\mathrm{VTI}}_{\mathrm{LVOT}}$ (SV = stroke volume; VTI = velocity time integral). When calculating the A_AV_, this principle is used to form the so-called (standard) continuity equation (CE_std_) as the ratio of constant flows through the LVOT and the following (smaller) aortic valve are inversely proportional to the corresponding areas, i.e., $A_{\mathrm{AV}}\cdot {\mathrm{VTI}}_{\mathrm{AV}}=A_{\mathrm{LVOT}}\cdot {\mathrm{VTI}}_{\mathrm{LVOT}}$, $A_{\mathrm{AV}}=\frac{A_{\mathrm{LVOT}}\;\cdot {\;\mathrm{VTI}}_{\mathrm{LVOT}}}{{\mathrm{VTI}}_{\mathrm{AV}}}$.

In clinical practice, the A_LVOT_ is calculated by measuring the anterior–posterior (a/p) diameter of the LVOT in the parasternal long axis only, thus assuming a round shape of the LVOT’s cross-sectional area, as described in the formula $A_{\mathrm{LVOT}}=\pi \cdot {\left(\frac{\mathrm{DiameterLVOT}}{2}\right)}^{2}$ [6]. However, it has been shown that the shape of the LVOT is eccentric, i.e., elliptical in shape, with the longer diameter extending from the medial–lateral axis (m/l), which is not recognized by 2D echocardiography [7]. In consequence, cardiac hemodynamics, and/or the anatomical aortic valve area, are underestimated [8,9,10]. 

This study aimed to assess multiple 2D and 3D measurements and calculations of the A_LVOT_ and A_AV_ in both TTE and TEE, and compare their performance and validity to the “gold standard” of the CT analysis.

## 2. Material and Methods

### 2.1. Patient Selection

We prospectively screened consecutive patients undergoing imaging for different clinical indications. Patients were included if both 3D-TTE and 3D-TEE were performed, as well as cardiac CT analysis. The study conformed to the Declaration of Helsinki. All patients provided written informed consent, and the study was approved by the local ethics committee (EK 111032015).

### 2.2. Echocardiography

**Figure 1:**
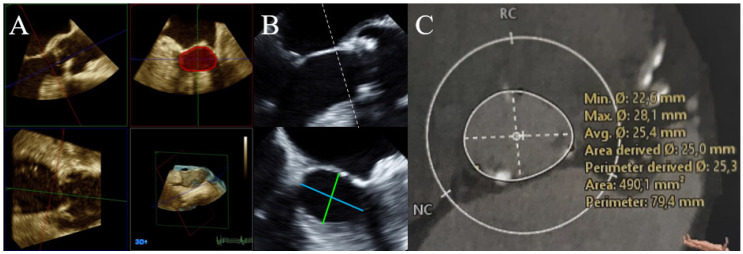
LVOT measurements, using different methods to measure LVOT parameters in one patient, as demonstrated with TEE (**A**,**B**) and CT (**C**). (**A**) 3D-volume MPR measurement of the ${3\mathrm{DA}}_{\mathrm{LVOT}}$ after proper alignment of the different cut-planes (green, blue, red). The transparent red area in the right upper quadrant measures 4.82 cm^2^. (**B**) Calculation of ${\mathrm{ellipticA}}_{\mathrm{LVOT}}$ ($=\pi \cdot \left(\frac{{\mathrm{DiameterLVOT}}_{a/p}\;\cdot {\;\mathrm{DiameterLVOT}}_{m/l}}{4}\right)=4.84{\;\mathrm{cm}}^{2}$ ) using the short a/p (green; 2.2 cm) and the long m/l diameters (turquoise; 2.8 cm). Calculation of ${\mathrm{stdA}}_{\mathrm{LVOT}}=\pi \cdot {\left(\frac{{\mathrm{DiameterLVOT}}_{a/p}}{2}\right)}^{2}=3.80{\;\mathrm{cm}}^{2}\;$. (**C**) Planimetry of ${\mathrm{CTA}}_{\mathrm{LVOT}}$ (transparent white, 4.901 cm^2^) using 3D-volume MPR. A_LVOT_—area of the LVOT, assessed using different methods; CT—computed tomography TEE—transesophageal echocardiography.

Imaging was acquired using Philips IE33 and EPIQ CVx ultrasound machines, equipped with either X5-1 TTE probes and X7-2t or X8-2t TEE probes. Analysis of the imaging data was carried out by two experienced echocardiographers (KS and TFR), using Philips QLAB (all Philips Health Systems N.V., Koninklijke, The Netherlands). First, A_LVOT_ was calculated using the standard approach, employing 2D-echocardiography assessment of the long axis, with ${\mathrm{stdA}}_{\mathrm{LVOT}}=\pi \cdot {\left(\frac{{\mathrm{DiameterLVOT}}_{a/p}}{2}\right)}^{2}$. Second, $3{\mathrm{DA}}_{\mathrm{LVOT}}$ was assessed using planimetry in multi-planar reconstruction (MPR) from 3D-echocardiography datasets. Third, to appreciate the elliptic form of the LVOT in a calculation, ${\mathrm{ellipticA}}_{\mathrm{LVOT}}=\pi \cdot \left(\frac{{\mathrm{DiameterLVOT}}_{a/p}\;\cdot {\;\mathrm{DiameterLVOT}}_{m/l}}{4}\right)$ was devised from 3D-biplane echocardiography, incorporating both a/p and m/l diameters of the LVOT (Figure 1A,B). Fourth, the standard CE_std_ was used to calculate ${\mathrm{stdA}}_{\mathrm{AV}}=\frac{{\mathrm{stdA}}_{\mathrm{LVOT}}\;\cdot {\;\mathrm{VTI}}_{\mathrm{LVOT}}}{{\mathrm{VTI}}_{\mathrm{AV}}}$. Fifth, $3{\mathrm{DA}}_{\mathrm{AV}}$ was measured using planimetry in MPR from 3D-echocardiography volumes. Finally, a modified continuity equation (CE_mod_) to appreciate the elliptic LVOT was used to calculate ${\mathrm{modA}}_{\mathrm{AV}}=\frac{{\mathrm{ellipticA}}_{\mathrm{LVOT}}\;\cdot {\;\mathrm{VTI}}_{\mathrm{LVOT}}}{{\mathrm{VTI}}_{\mathrm{AV}}}$. Measurements and calculations were performed in both TTE and TEE. 

### 2.3. CT

CT measurements served as the “gold standard” comparator to the echocardiographic measurements and calculations. Using planimetry in MPR, variables measured by CT were ${\mathrm{CTA}}_{\mathrm{LVOT}}$ (Figure 1C) and ${\mathrm{CTA}}_{\mathrm{AV}}$. Analysis of the data was carried out by two experienced specialists (KS and TFR), using 3mensio structural heart (3mensio Medical Imaging, Bilthoven, The Netherlands). Imaging was acquired using Siemens SOMATOM Force (Siemens Healthineers, Erlangen, Germany). 

### 2.4. Statistical Analysis

Results are represented as median with interquartile range (IQR [Q1, Q3]) or frequencies with percentages (%). 

The comparisons of values included correlation and Bland–Altman analyses to assess the congruence using bias and limits of agreement between the different techniques [11]. Normality distribution was tested using the Kolmogoroff–Smirnoff test. Bias between measurements was assessed using a paired or unpaired Student’s *t*-test, or a Wilcoxon signed-rank test, as appropriate. Differences between parameters for multiple groups was assessed by analysis of variance or by the Kruskal–Wallis test, as appropriate. Statistical significance was defined as a two-tailed *p*-value < 0.05. Significance criteria in multiple testing were adjusted by the Bonferroni method. All analyses were performed using R (version 3.0.2, 2013, The R Foundation for Statistical Computing, Vienna, Austria).

## 3. Results

From 2 January 2017 to 28 December 2018, 421 patients were examined. Complete data were available for 107 patients (Supplementary Figure S1). Baseline characteristics showed a median age of 74 years, and a higher prevalence of the male sex (55%). Further details, including comorbidities, are shown in Table 1.

Left ventricular dimensions showed normal median ranges in diastole (LVEDD 47 mm [42, 54]) and systole (LVESD 32 mm [25, 40]). Median LVEF was 57% [41, 65]. No or mild aortic stenosis (AS) was present in 50% of cases, while moderate AS was present in 10%, and severe AS in 40% of cases (Table 2).

While the a/p diameters of the LVOT did not differ between imaging modalities (*p* = 0.98), the m/l dimensions in the 3D biplane were smaller in TEE (2.53 cm [2.3, 2.77]) and larger in TTE (2.67 [2.38, 2.95]), when compared to CT (2.59 [2.42, 2.90]; *p* = 0.002). When comparing the two dimensions of the LVOT, a/p diameters were significantly smaller than m/l diameters in all 3D-TTE (2.02 cm [1.90, 2.16] vs. 2.67 cm [2.38, 2.95]; *p* < 0.001), 3D-TEE (2.02 cm [1.92, 2.13] vs. 2.53 cm [2.30, 2.77]; *p* < 0.001), and CT (2.04 cm [1.90, 2.13] vs. 2.59 cm [2.42, 2.90]; *p* < 0.001). Correspondingly, the eccentricity index, defined as the ratio of a/p to m/l diameters, was larger in the 3D-TEE assessment (0.79 [0.75, 0.84]), and smaller in the 3D-TTE evaluation (0.75 [0.70, 0.82]), when compared to CT (0.76 [0.71, 0.81]; *p* < 0.001). 

**Figure 2:**
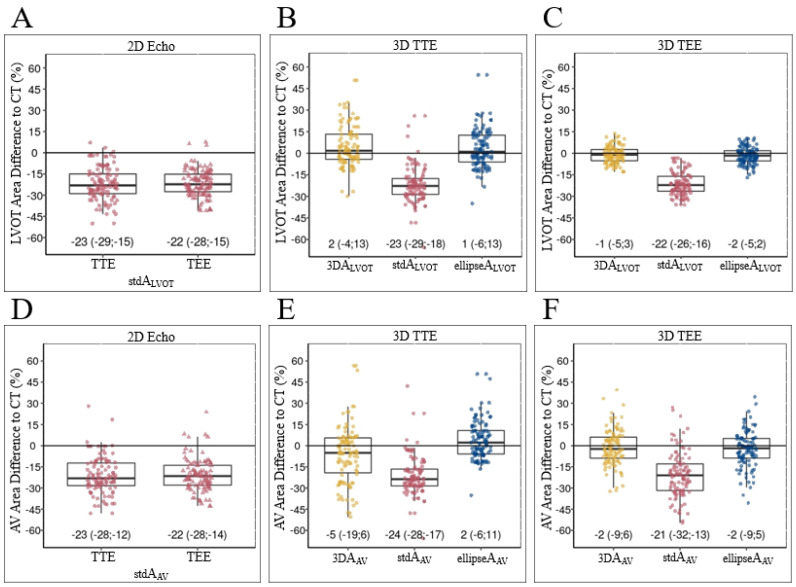
Different LVOT and AV area measurements. Boxplots showing the different echo measurements of LVOT (**A**–**C**) and AV areas (**D**–**F**) compared to CT (solid center 0 line in each field). (**A**) Both 2D-TTE and 2D-TEE showed > 20% underestimation of LVOT area when using ${\mathrm{stdA}}_{\mathrm{LVOT}}$ calculation. (**B**,**C**) Both 3D-TTE and 3D-TEE showed > 20% underestimation of LVOT area when using ${\mathrm{stdA}}_{\mathrm{LVOT}}$ calculation (middle column), while each $3{\mathrm{DA}}_{\mathrm{LVOT}}$ (left column) and ${\mathrm{ellipseA}}_{\mathrm{LVOT}}$ (right column) showed good congruence with CT. Measurements and calculations in 2D echo (**D**) and 3D echo (**E**,**F**) showed similar results for A_AV_. A_AV_—area of the aortic valve; A_LVOT_—area of the LVOT (assessment for both AAV and ALVOT given in Figure 1); CT—computed tomography; TEE—transesophageal echocardiography; TTE—transthoracic echocardiography.

Calculating ${\mathrm{stdA}}_{\mathrm{LVOT}}$ from the a/p dimension yielded more than 20% lower values in all echocardiography assessments compared to CT (*p* < 0.001). Both 3D-volume MPR (*p* = 0.14) and use of the novel equation ${\mathrm{ellipticA}}_{\mathrm{LVOT}}\;$(*p* = 0.20) showed no significant difference (Figure 2A–C). Details of the comparisons of LVOT measurements by the different imaging methodologies are summarized in Table 3.

**Figure 3:**
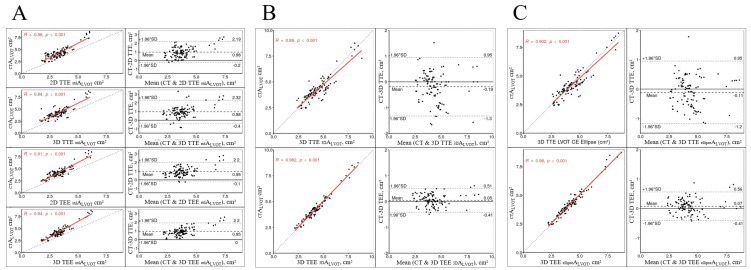
Scatter plots (left, correlation) and corresponding Bland–Altman plots (right, congruence) of different analyses of A_LVOT_. In general, correlation was good in all measurements. (**A**) Compared to CT, use of CE_std_ showed consistent underestimation of A_LVOT_ with poor congruence, irrespective of echocardiography modality. (**B**) Ascertainment of A_LVOT_ using 3D planimetry showed good congruence that improved when using 3D TEE. (**C**) Calculation of A_LVOT_ using CE_mod_ also showed good congruence to CT, with superior assessments in 3D TEE. A_LVOT_—area of the LVOT (for assessment methods, see Figure 1); CT—computed tomography; TEE—transesophageal echocardiography; TTE—transthoracic echocardiography.

All echocardiographic measurements correlated significantly and strongly with CT (all *p* < 0.001; Figure 3). Congruence of ${\mathrm{stdA}}_{\mathrm{LVOT}}$ measurements with “gold standard” of CT was low, irrespective of TTE or TEE. In contrast, 3D-volume MPR and ${\mathrm{ellipticA}}_{\mathrm{LVOT}}\;$showed high congruence with CT (Figure 3). The strongest correlations and congruence were found in TEE when using either 3D planimetry for ${\mathrm{ellipticA}}_{\mathrm{LVOT}}$ (R = 0.982, *p* < 0.001; bias = 0.05, LOA: −0.41; 0.51), or 3D biplane for ${\mathrm{ellipticA}}_{\mathrm{LVOT}}$ (R = 0.980, p < 0.001; bias = 0.07, LOA: −0.41, 0.56). The least correlation and congruence showed TTE for ${\mathrm{stdA}}_{\mathrm{LVOT}}$ in either 2D- (R = 0.880, 0.001; bias = 0.98; LOA: −0.2, 2.19), or 3D-biplane measurements (R = 0.840, *p* < 0.001; bias = 0.98, LOA: −0.4, 2.32). 

Regarding the AV, where CE_std_, CE_mod_, and 3D-volume MPR were used to receive the A_AV_, results are in principle similar to LVOT assessments. The ${\mathrm{stdA}}_{\mathrm{AV}}$ showed smaller values than the ${3\mathrm{DA}}_{\mathrm{AV}}$ and ${\mathrm{ellipticA}}_{\mathrm{AV}}$ measurements (Table 4, Figure 2D–F). The lowest bias and the best congruence were found in TTE using 3D-biplane ${\mathrm{modA}}_{\mathrm{AV}}$ (bias 0.06, LOA: −0.45, 0.56) and in TEE using both 3D-volume MPR for $3{\mathrm{DA}}_{\mathrm{AV}}$ (bias −0.03, LOA: −0.38, 0.32) and 3D biplane for ${\mathrm{modA}}_{\mathrm{AV}}$ (bias −0.04, LOA: −0.4, 0.32). The highest bias and least congruence were seen using any ${\mathrm{stdA}}_{\mathrm{AV}}$ (Supplementary Figure S2).

## 4. Discussion

Accurate measurement of the A_LVOT_ is pivotal to correctly calculating left cardiac hemodynamic states and/or the A_AV_ to assess the severity in AS, using the continuity equation. Our study offers comparison of a wide spectrum of different echocardiographic imaging methods to the “gold standard” of CT data as a reference. The principles used to receive A_LVOT_ and A_AV_ were either calculation (${\mathrm{stdA}}_{\mathrm{LVOT}}$ and ${\mathrm{ellipticA}}_{\mathrm{LVOT}}$; ${\mathrm{stdA}}_{\mathrm{AV}}$ and ${\mathrm{modA}}_{\mathrm{AV}}$), or planimetry using MPR ($3{\mathrm{DA}}_{\mathrm{LVOT}}$ and $3{\mathrm{DA}}_{\mathrm{AV}})$. The echocardiographic methods employed were common 2D echocardiography (${\mathrm{stdA}}_{\mathrm{LVOT}}$ and ${\mathrm{stdA}}_{\mathrm{LVOT}}$), 3D-biplane interrogation (${\mathrm{ellipticA}}_{\mathrm{LVOT}}$ and ${\mathrm{modA}}_{\mathrm{AV}}$), and 3D-volume planimetry ($3{\mathrm{DA}}_{\mathrm{LVOT}}$ and $3{\mathrm{DA}}_{\mathrm{AV}})$, in both TTE and TEE, respectively. 

In this study, we have shown that first, the A_LVOT_ underestimated the anatomic area when the standard echo calculation was used, compared to CT. This was irrespective of the echocardiography approach used. Second, $3{\mathrm{DA}}_{\mathrm{LVOT}}$ assessed using planimetry in 3D echocardiography showed excellent congruence and correlation with CT measurements for both 3D-TTE and 3D-TEE. 3D-TEE was superior to 3D-TTE in both congruence and correlation. Third, the adapted formula to calculate an elliptic LVOT’s anatomy, ${\mathrm{ellipticA}}_{\mathrm{LVOT}}$, also showed both excellent correlation and congruence with CT measurements for both 3D-TTE and 3D-TEE. Again, 3D-TEE was superior to 3D-TTE in both congruence and correlation. Fourth, using the CE_std_ leads to underestimating the anatomic A_AV_, irrespective of the echocardiography approach used. Fifth, while $3{\mathrm{DA}}_{\mathrm{AV}}$ showed excellent correlation to CT for both 3D-TTE and 3D-TEE, congruence of 3D-TEE measurements was conspicuously superior. Finally, using the adapted ${\mathrm{ellipticA}}_{\mathrm{LVOT}}$ in the CE_mod_ showed ${\mathrm{modA}}_{\mathrm{AV}}$ also had excellent correlation and congruence with CT measurements for both 3D-TTE and 3D-TEE—again with superior performance of 3D-TEE. 

Our findings of underappreciating both the A_LVOT_ and A_AV_ when using the standard echocardiographic interrogation technique are in line with current data. Different methods have been proposed for correcting this flaw, e.g., using a fixed correction factor [12], evaluating the aortic annulus instead of the LVOT, as it becomes "more" round in shape during systole [13], or using the A_LVOT_ as directly measured using CT or 3D-TTE planimetry [10,14]. As the LVOT comes in different shapes and sizes, including round-based LVOTs, a fixed correction factor, while maybe statistically correct, seems less appropriate for the assessment of the individual patient. Then, the data suggesting that the aortic annulus becomes "more" round in shape during systole have been challenged by other studies, which rather suggests that the aortic annulus is just as elliptical as the LVOT [15,16,17]. Furthermore, correct placement of the pulsed-wave Doppler (PWD) sample volume to acquire the appropriate measurements of the velocity (and thus gradient), as well as the velocity time integral of the flow, is highly challenging when the aortic annulus is chosen as the point of interrogation: since the aortic annulus moves up and down during the cardiac cycle, the sample volume of the PWD will not stay at the correct site. Hence, there is a very high risk of either measuring the speed at the level of the LVOT (velocities may be lower), or at the level of the (stenotic) aortic valve (thus yielding much higher velocities), instead of the aortic annulus. Concerning planimetry of the LVOT using 3D data, our results support the assumption that its use from MPR in either 3D echocardiography or CT seems to be a more accurate approach. However, even though echocardiography is widely available, 3D-MPR analysis is not. This is caused by multiple factors: Not all echo machines can acquire 3D volumes. If 3D volumes can be obtained, analysis is dependent on specific (and often expensive) software, e.g., QLAB, EchoPac (GE Healthcare, Chicago, IL, USA), TOMTEC (TOMTEC Imaging Systems GmbH, Unterschleissheim, Germany), 3Mensio, and others. Finally, if these requirements can be fulfilled, 3D-volume analysis using MPR requires special training and is sometimes not feasible [18]. On the other hand, 3D biplane is much more available, has both a higher spatial and temporal resolution than 3D-volume imaging, and assessment is very similar to the 2D imaging physicians are used to. Our data also show 3D-TEE is superior to all other echocardiographic approaches. However, the use of both the ${\mathrm{ellipticA}}_{\mathrm{LVOT}}$ and the CE_mod_, yielding the ${\mathrm{modA}}_{\mathrm{AV}}$, offer attractive alternatives, even if they are based on the more available 3D-biplane interrogation and TTE, respectively. Circumventing 3D evaluation in favor of direct 2D-TTE planimetry in the parasternal short axis of either the A_LVOT_ and/or the A_AV_ should be discouraged. First, oblique cut-planes, and second, lower image quality of TTE—especially relevant in calcified AS—can easily lead to incorrect measurements [19,20].

Recent guidelines still advocate the use of standard echocardiographic interrogation to assess and grade AS, although the problem of underestimating both the A_LVOT_ and A_AV_ is well-known [6,21]. The reason is that despite assuming a circular LVOT shape in the continuity equation, use of this calculation has shown to be a strong predictor of clinical outcomes. This suggests that aortic stenosis might be of relevance earlier than a progression of the anatomic A_AV_ below 1 cm^2^. This is somewhat backed by the observation that treatment of asymptomatic patients with severe AS shows a mortality benefit [22]. Hemodynamics seem even more complex in patients with moderate AS and reduced LVEF, prompting the TAVR UNLOAD trial [23,24,25]. In this light, use of a CE_std_ yielding imprecise results hardly seems satisfying—scientifically and clinically. Anatomically correct echocardiographic assessment and grading of the underlying disease is a necessity void of compromises.

### Study Limitations

As this study was held at one center only, the external validity might be questionable. For instance, differences in personnel and logistics in general and differences in TTE, and TTE operator experience in particular, can cause wide variations on the outcome. However, there were only a limited number of highly qualified TTE and TEE operators, following the same protocol during the examinations. Additionally, the data were re-evaluated by two blinded cardiologists, both experts in echocardiography. With the single-center character of the study, a selection bias cannot be ruled out. However, as the study population was an “all comers” selection, comprised of patients with a very heterogeneous disease spectrum, this seems less likely.

Differences in imaging quality were not addressed specifically. Image quality in the study population was generally good. Decreased image quality will most likely lead to different results. In these cases, CT angiography analysis might prove to be superior to echocardiography.

## 5. Conclusions

The LVOT area, as well as the aortic valve area, is systematically underestimated when using standard calculations based on 2D-echocardiography interrogations, due to the eccentricity of the LVOT. The use of 3D echocardiography is significantly superior due to its good correlation and its data being congruent with CT analysis. The use of adapted formulas designed to recognize the elliptic LVOT shape also yielded excellent correlation and congruence with CT data, with the underlying echocardiographic method of 3D biplane being widely available and easy to use.

## Figures and Tables

**Table 1:** Baseline characteristics.

Characteristic	n = 107
Age, years	74 [64, 80]
Male sex	59 (55)
NYHA class	
0	19 (18)
I	17 (16)
II	28 (26)
III	32 (30)
IV	11 (10)
CAD	55 (51)
aHT	93 (87)
D.m. II	42 (39)
HLP	59 (55)
Obesity	48 (45)

aHT—arterial hypertension, CAD—coronary artery disease, D.m. II—type 2 diabetes mellitus, HLP—hyperlipoproteinemia, NYHA—New York Heart Association. Values are n (%), or median [Q1; Q3].

**Table 2:** Baseline echocardiographic characteristics.

Characteristic	n = 107
LV EDD, mm	47 [42, 54]
LV ESD, mm	32 [25, 40]
IVSD, mm	13.0 [10.9, 14.1]
LV EF, %	57 [41, 65]
Vmax AV, cm/s	321 [148, 415]
Maximum PG AV, mm Hg	40 [9; 69]
Mean PG AV, mm Hg	23 [5, 40]
VTI AV, cm	51 [27, 86]
Vmax LVOT, cm/s	89 [72, 100]
VTI LVOT, cm	19 [15, 25]
AS grade	
0	52 (49)
I	1 (1)
II	11 (10)
III	43 (40)

AS grade—aortic valve stenosis grade (grading as calculated by standard echocardiographic approach); AV—aortic valve; EDD—end-diastolic diameter; ESD—end-systolic diameter; EF—ejection fraction; IVSD—inter-ventricular septum thickness in diastole; LV—left ventricular; LVOT—left ventricular outflow tract; PG—pressure gradient; Vmax—peak velocity; VTI—velocity time integral. Values are n (%), or median [Q1; Q3].

**Table 3:** Comparison of LVOT measurements by the different imaging methodologies.

LVOT	2D-TTE	3D-TTE	2D-TEE	3D-TEE	CT MPR	*p*-Value
a/p dimension (cm)	2.01 [1.86, 2.17]	2.02 [1.90, 2.16]	2.00 [1.89, 2.17]	2.02 [1.92, 2.13]	2.04 [1.90, 2.13]	0.98
m/l dimension (cm)		2.67 [2.38, 2.95]		2.53 [2.30, 2.77]	2.59 [2.42, 2.90]	0.002
eccentricity (=a/p: m/L)		0.75 [0.70, 0.82]		0.79 [0.75, 0.84]	0.76 [0.71, 0.81]	<0.001
${\mathrm{stdA}}_{\mathrm{LVOT}}$(cm^2^)	3.18 [2.74, 3.70]	3.17 [2.84, 3.66]	3.17 [2.82, 3.68]	3.23 [2.87, 3.58]	4.15 [3.63, 4.53]	<0.001
${3\mathrm{DA}}_{\mathrm{LVOT}}$(cm^2^)		4.22 [3.64, 5.06] *		4.11 [3.59, 4.61] *		0.14
${\mathrm{ellipticA}}_{\mathrm{LVOT}}$(cm^2^)		4.24 [3.57, 4.92]		4.00 [3.49, 4.52]		0.20

2D—two-dimensional; 3D—three-dimensional; TEE—transesophageal echocardiography; CT—computed tomography; LVOT—left ventricular outflow tract; MPR—multi-planar reconstruction; TTE—transthoracic echocardiography; values are median [Q1; Q3]. *p*-value denotes the non-parametric Kruskal–Wallis test for multiple samples. In 3D echocardiography, values were measured in either 3D biplane or in 3D volumes using MPR *.

**Table 4:** AVA (cm^2^) derived from different echocardiographic methods compared to CT-MPR.

Echocardiographic Method	Bias	LOA (Lower; Upper)
**TTE**		
$2D\;\mathrm{standard}\;({\mathrm{stdA}}_{\mathrm{AV}}$)	−0.4 ^†^	−1; 0.21
$3D-\mathrm{biplane}\;\mathrm{standard}\;({\mathrm{stdA}}_{\mathrm{AV}}$)	−0.41 ^†^	−1.13; 0.31
$3D-\mathrm{volume}\;\mathrm{MPR}\;({3\mathrm{DA}}_{\mathrm{AV}}$)	−0.07 *¶	−0.71; 0.56
$3D-\mathrm{biplane}\;\mathrm{ellipse}\;({\mathrm{ellipticA}}_{\mathrm{AV}}$)	0.06 *¶	−0.45; 0.56
**TEE**		
$2D\;\mathrm{standard}\;({\mathrm{stdA}}_{\mathrm{AV}}$)	−0.4 ^†^	−0.96; 0.16
$3D-\mathrm{biplane}\;\mathrm{standard}\;({\mathrm{stdA}}_{\mathrm{AV}}$)	−0.41 ^†^	1.05; 0.24
$3D-\mathrm{TEE}\;\mathrm{planimetry}\;({3\mathrm{DA}}_{\mathrm{AV}}$)	−0.03 *¶	−0.38; 0.32
$3D-\mathrm{biplane}\;\mathrm{TEE}\;({\mathrm{ellipticA}}_{\mathrm{AV}}$)	−0.04 *¶	−0.4; 0.32

2D—two-dimensional; 3D—three-dimensional; TEE—transesophageal echocardiography; CT—computed tomography; LOA—limit of agreement; LVOT—left ventricular outflow tract; MPR—multi-planar reconstruction; TTE—transthoracic echocardiography; values are median [Q1; Q3]. *p*-value denotes the non-parametric Kruskal–Wallis test for multiple samples. ^†^ = *p* < 0.001 when compared to CT-MPR; * = *p* < 0.001 for MPR planimetry or ellipse method was compared to classic method.

## Supplementary Materials

The following are available online at https://www.mdpi.com/article/10.3390/diagnostics12071656/s1.
