# Supplementary material for: Calculation of Aortic VAlve and LVOT Areas by a Modified Continuity Equation Using Different Echocardiography Methods: The CAVALIER Study
Source: Diagnostics (Basel). 2022 Jul 7;12(7):1656. doi: 10.3390/diagnostics12071656 (PMC9321790; doi:10.3390/diagnostics12071656)
Supplement: Supplementary file 1 [file diagnostics-12-01656-s001.zip › diagnostics-1771605-supplementary.pdf]

Supplementary Figure Legends

Supplementary Figure S1–Patient Flowchart

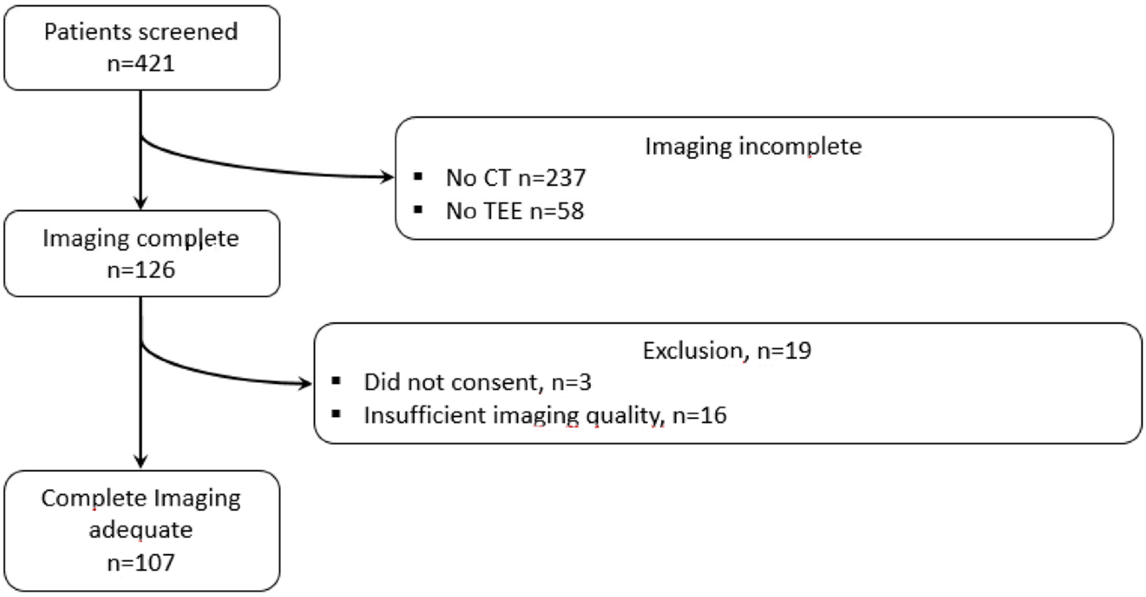

CT–Computed Tomography; TEE–Transesophageal Echocardiography

Supplementary Figure S2

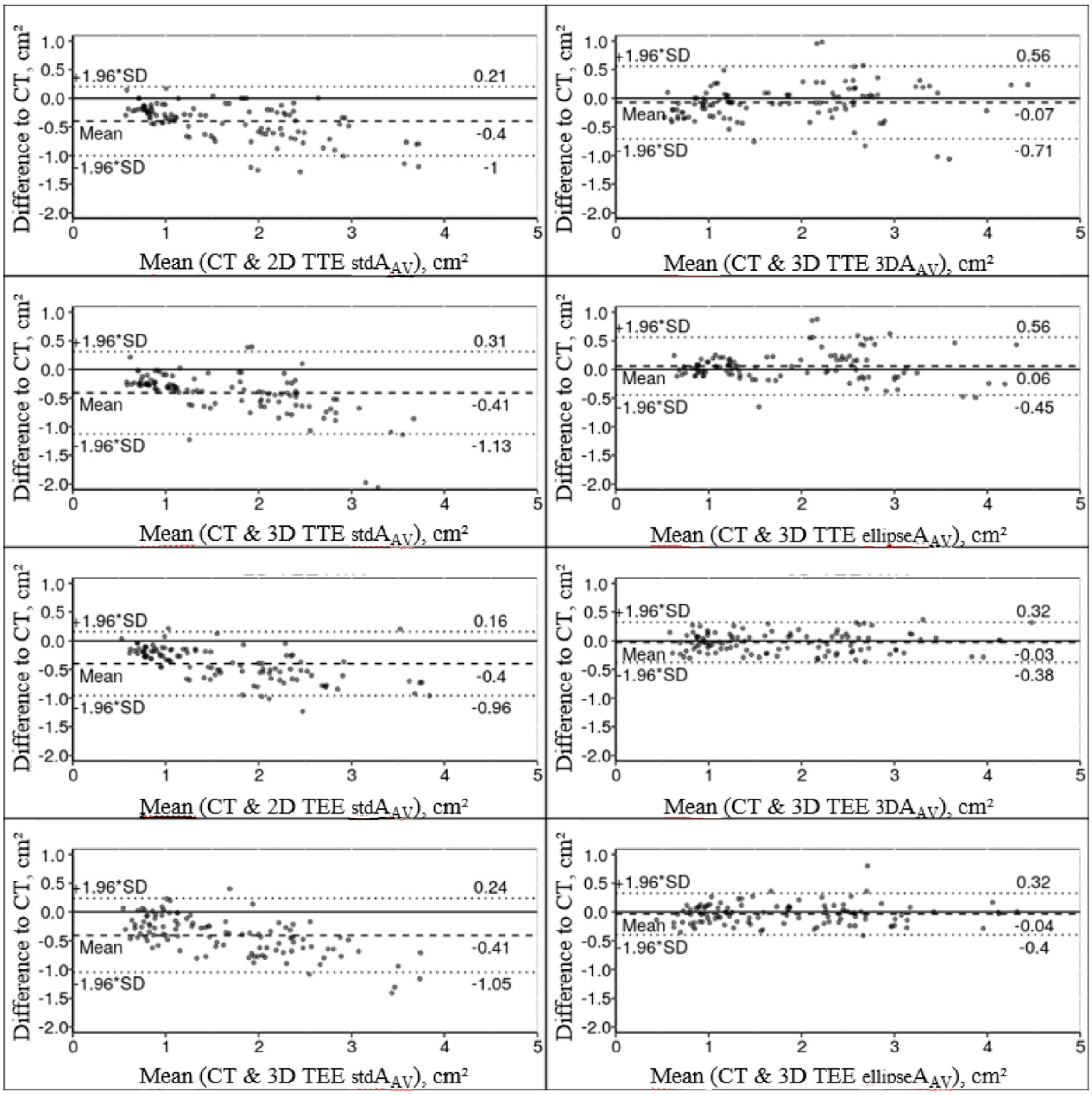

Bland-Altman plots (congruence) of different analyses of A_{AV}. In general, correlation was good in all measurements. (A) Compared to CT, use of CE_{std} shows consistent under-estimation of A_{LVOT} with poor congruence, irrespective of echocardiography modality. (B) Ascertainment of A_{LVOT} using 3D planimetry shows good congruence that is better when using 3D TEE. (C) Calculation of A_{AV} using CE_{mod} also shows good congruence to CT, with superior assessments in 3D TEE.

A_{AV} – Area of the aortic valve (assessment methods for both AAV and ALVOT see figure 1); CT

– Computed Tomography; TEE – Transesophageal Echocardiography; TTE – Transthoracic Echocardiography.
